# How Useful Are International Treatment Guidelines in Low- and Middle-Income Countries?

**DOI:** 10.1200/JGO.2016.008250

**Published:** 2017-01-18

**Authors:** Stewart Kerr, Abdul-Rahman Jazieh, David Kerr

**Affiliations:** **Stewart Kerr** and **David Kerr**, Africa Oxford Cancer Foundation, Oxford, United Kingdom; and **Abdul-Rahman Jazieh**, King Saud bin Abdulaziz University for Health Sciences, Riyadh, Kingdom of Saudi Arabia.

One of the recent growth industries and, one might add, great successes in global oncology has been the rise of international treatment guidelines. The most prominent of these guidelines include those by ASCO,^1^ the National Comprehensive Cancer Network (NCCN),^2^ and the European Society of Medical Oncology (ESMO),^3^ which provide timely, evidence-based recommendations for the multidisciplinary management of most cancer types. They provide an international gold standard in a fairly user-friendly format, tend not to be overly proscriptive when evidence favors neither one nor another regimen, and are backed by well-respected experts who provide additional data on the depth of evidence to support a particular recommendation. International treatment guidelines are updated regularly, which is important when the standard of care may change from quarter to quarter and can provide a point of reference for hard-pressed clinicians. Until recently, only the gold standard existed, with no mention of silver, bronze, or tin! One wonders what fraction of the world’s cancer community might be supported by a health care system that could afford full and undiluted access to gold standard care, perhaps on the order of 10%?

An African health minister might have $10 per head of his or her population to spend on all of health care, let alone cancer, whereas a conservative estimate of cancer spending is approximately $150 million per million population in the developed world.^4^ How then can these trusted guidelines serve oncologists who work in low- and middle-income countries (LMICs)?

We conducted an online survey of oncologic practice with respect to the treatment of lung and breast cancer in LMICs including India, China, Thailand, the Philippines, Malaysia, Vietnam, Indonesia, Argentina, Brazil, Chile, and Mexico. The majority of survey respondents were consultants (44%) or oncology department chiefs (31%) from university hospitals (48%) and general private hospitals (35%); the other respondents were from community hospitals. These respondents, therefore, represent a senior and influential group of oncologists who are likely considered key opinion leaders in their countries. The full data set will be published, but significant international differences in the use of specific regimes in defined disease settings exist.

Of the 139 respondents, 58% claimed to always use guidelines (often different ones for different diseases) to support their clinical decisions. The guidelines used vary, with some referring to more than one set of guidelines. Ninety-two percent use NCCN guidelines, 55% ASCO guidelines, 55% ESMO guidelines, and 40% national guidelines. All respondents mainly rely on NCCN guidelines, predominantly for private and self-pay patients, because national health care systems and insurance coverage are not sufficiently funded to support particular cancer treatment protocols. Of the respondents who use national guidelines, their stated reason for not relying on the international guidelines is that the treatments specified in international guidelines are not easily accessible within their countries. Seventy-five percent of respondents who use international guidelines modify them in some way to treat their patients, which contrasts with only 50% who rarely have to modify national guidelines.

We do not have a one-size-fits-all single set of guidelines of universal applicability but rather a pick-and-mix approach that dips into various guidelines according to disease, stage, affordability, and whether the oncologist was trained in the United States or Europe. At one level, this approach is not surprising: Can uniformity of cancer treatment reside as a universal verity, as argued by Plato, or, rather, as shades of opinion and interpretation?

These data suggest that the international guidelines groups could take two utilitarian steps to increase their usefulness outside the United States or western Europe, namely by consulting with clinicians within a geographic region on how their guidelines might be better adapted to serve the local community of physicians and their patients and to introduce an element of resource stratification derived from the concept of affordability.^5^ ESMO and ASCO both have international input into their clinical guideline committees, but NCCN has taken a step farther to globalize its advice by hosting regional conferences in which invited senior clinicians modify, adapt, and customize the parent guideline set. Because the majority of clinical trial evidence that support guidelines is still generated in the West in predominantly white patients, these data probably will always be borrowed until a sufficient regional clinical trials infrastructure permits stronger regional trial recruitment. Nevertheless, this experiential approach suffices as a temporary bridge across continents, cultures, and ethnicities.

Medicine affordability is a major barrier to cancer drug access, and cancer generally is acknowledged as the most common disease associated with medical bankruptcy. The term financial toxicity is frequently used by the public and policymakers with regard to cancer treatment, which has led both ASCO and NCCN to include some estimate of affordability in their guidance.

The NCCN Evidence Blocks are an easily accessible visual representation of five key components of value that provide important information about specific recommendations contained within the NCCN Clinical Practice Guidelines. These five components are efficacy, safety, quality and quantity of evidence, consistency of evidence, and affordability. ASCO’s Value Framework^6^ has been constructed as a conceptual model that incorporates the elements of clinical benefit, toxicity, and symptom palliation as derived from a comparative clinical trial and combines these elements into a score termed the net health benefit. Information on the cost of the regimens also will be presented so that the patient can consider the relative financial impact of the treatment options, an essential component of delivering cancer care in LMICs.

Many would argue that a single, global, evidence-based standard of care for patients with cancer should exist and that to detract or divert from this standard is a breach of human rights. Such an argument accepts that the world will always be riven by inequity between the haves and have nots and that to support cancer treatment with anything less than ideal is to promulgate this base philosophy. We regard this argument as wholly specious and subscribe to the philosophy that the perfect is the enemy of the good. Guidance should not be prevented from being offered where certain diagnostic tests and treatment approaches are unavailable, and NCCN has taken the lead to define appropriate treatment pathways that are based on available resources. Basic, Core, and Enhanced Resources and NCCN Guidelines identify treatment options that will provide the best possible outcomes given specific resource constraints.^7^ The NCCN Framework resources are defined as follows:

Basic Resources: essential services needed to provide a minimal standard of care.
Core Resources: resources provided in the Basic Resources Framework plus services that provide major improvements in disease outcomes (eg, survival) and that are not cost prohibitive.
Enhanced Resources: resources provided in the Core Resources Framework plus services that provide lesser improvements in disease outcomes and/or services that provide major improvements in disease outcomes but are cost prohibitive in lower-resource settings.
NCCN Guidelines: resources provided in the Enhanced Resources Framework plus services that provide minor improvements in disease outcomes, interventions that are cost prohibitive in lower-resource settings, and/or services that do not provide improvement in disease outcomes but are desirable.

ASCO has published two resource-stratified guidelines in the *Journal of Global Oncology* for cervical cancer screening and treatment.^8,9^ A multidisciplinary, multinational panel of cancer control, medical and radiation oncology, health economics, obstetric and gynecologic, and palliative care experts produced recommendations that reflect resource-tiered settings. Existing sets of guidelines were identified and reviewed, and adapted recommendations form the evidence base for clinicians and patients in relatively impoverished situations.

Everyone involved in guideline production believes that the best available resources should be delivered. However, if basic resources for cancer treatment are unavailable, palliative and best supportive care should be provided. These tiered guidelines can also be used to inform health policy and national cancer plans in that they define a minimal baseline of resources required to establish foundation levels of cancer treatment.

In summary, good evidence suggests that several of the issues highlighted in our survey of colleagues who work in LMICs are being addressed by the major professional societies and guidelines groups, namely, adaptation for local use by engaging with clinicians in specific geographic regions, stratifying resources and defining minimal treatment standards, engaging with the concept of value, and providing tools to enable the often difficult discussion about treatment affordability between the patient and the physician.
